# Arbuscular Mycorrhizas Regulate Photosynthetic Capacity and Antioxidant Defense Systems to Mediate Salt Tolerance in Maize

**DOI:** 10.3390/plants9111430

**Published:** 2020-10-24

**Authors:** Hao Wang, Liyan Liang, Baoxing Liu, Di Huang, Shuo Liu, Runjin Liu, Kadambot H.M. Siddique, Yinglong Chen

**Affiliations:** 1 State Key Laboratory of Soil Erosion and Dryland Farming on the Loess Plateau, Institute of Soil and Water Conservation, Northwest A & F University, and Chinese Academy of Sciences, Yangling 712100, Shaanxi, China; hao_wang@nwafu.edu.cn (H.W.); liyan@nwafu.edu.cn (L.L.); lbx@nwafu.edu.cn (B.L.); huang-di@nwafu.edu.cn (D.H.); liushuo@nwafu.edu.cn (S.L.); 2 The University of Chinese Academy of Sciences, Beijing 100049, China; 3 Institute of Mycorrhizal Biotechnology, Qingdao Agricultural University, Qingdao 266109, Shandong, China; liurj@qau.edu.cn; 4 The UWA Institute of Agriculture, & School of Agriculture and Environment, The University of Western Australia, Perth, WA6001, Australia; kadambot.siddique@uwa.edu.au

**Keywords:** arbuscular mycorrhizal fungi, salinity, oxidative damage, gas exchange, chlorophyll fluorescence

## Abstract

Salt stress inhibits photosynthetic process and triggers excessive formation of reactive oxygen species (ROS). This study examined the role of arbuscular mycorrhizal (AM) association in regulating photosynthetic capacity and antioxidant activity in leaves of two maize genotypes (salt-tolerant JD52 and salt-sensitive FSY1) exposed to salt stress (100 mM NaCl) in soils for 21 days. The leaf water content, chlorophyll content, and photosynthetic capacity in non-mycorrhizal (NM) plants were decreased by salt stress, especially in FSY1, with less reduction in AM plants than NM plants. Salinity increased the activities of antioxidant enzymes (superoxide dismutase (SOD), catalase (CAT), ascorbate peroxidase (APX), and glutathione reductase (GR)) in both genotypes regardless of AM inoculation, but decreased the contents of non-enzymatic antioxidants (reduced glutathione (GSH) and ascorbate (AsA)), especially in FSY1, with less decrease in AM plants than NM plants. The AM plants, especially JD52, maintained higher photosynthetic capacity, CO_2_ fixation efficiency, and ability to preserve membrane integrity than NM plants under salt stress, as also indicated by the higher antioxidant contents and lower malondialdehyde (MDA)/electrolyte leakage in leaves. To conclude, the higher salt tolerance in AM plants correlates with the alleviation of salinity-induced oxidative stress and membrane damage, and the better performance of photosynthesis could have also contributed to this effect through reduced ROS formation. The greater improvements in photosynthetic processes and antioxidant defense systems by AM fungi in FSY1 than JD52 under salinity demonstrate genotypic variation in antioxidant defenses for mycorrhizal amelioration of salt stress.

## 1. Introduction

Soil salinization is an environmental constraint that perturbs plant growth and metabolism, which is worsening with unsustainable cultivation practices and global warming. Arbuscular mycorrhizal (AM) fungi are beneficial soil microorganisms that live in symbiosis with most land plants. Inoculation with AM fungi can alleviate growth inhibition and salinity effects in both halophytes and glycophytes [1,2,3].

Salt toxicity lowers photosynthetic capacity and water status in plant tissues, leading to a decline in productivity [4,5]. Sheng et al. [6] reported that AM colonization improved maize photosynthetic capacity by increasing plant water status and regulating energy bifurcation between photochemical and non-photochemical events. Hajiboland et al. [3] attributed the influence of the AM symbiosis on tomato photosynthetic activity to increased stomatal conductance and photosystem II (PSII) efficiency. Porcel et al. [2] demonstrated that the higher photochemical efficiency for CO_2_ fixation and light energy utilization in AM rice plants facilitated salinity tolerance, which translated into higher photosynthetic and rubisco activities. However, limited studies have investigated the effect of AM fungi on leaf photosynthetic properties under saline conditions.

Like other abiotic stresses, salt imposes secondary oxidative stress on plants [7]. Oxidative stress occurs when multiple metabolic pathways in plants are uncoupled and high rates of electrons or energy transferred to molecular oxygen to generate reactive oxygen species (ROS) [7]. Toxic ROS involve the superoxide anion ($O_{2}^{•-}$), hydroxyl radical (${\mathrm{OH}}^{•}$), hydrogen peroxide (H_2_O_2_), and singlet oxygen (^1^O_2_), which are capable of disrupting normal metabolism. The ROS accumulate in leaves, causing peroxidation of cellular molecules like membrane lipids, protein, and nucleic acids [8], and targeting against photosynthetic apparatus and pigments [9]. Plants evolve detoxification systems to combat oxidative damage [8]. The prime mechanism for detoxifying ROS is the induction of antioxidant defense systems [10], which comprise a range of functionally interrelated enzymatic and non-enzymatic antioxidants for salt tolerance. Antioxidant enzymes include superoxide dismutase (SOD), catalase (CAT), ascorbate peroxidase (APX), and glutathione reductase (GR). SOD converts $O_{2\;}^{-}\;$to H_2_O_2_, APX and CAT convert H_2_O_2_ to H_2_O and O_2_, and GR is an integral component of the ascorbate-glutathione pathway that is responsible for removing H_2_O_2_ in different cellular compartments [11]. Non-enzymatic antioxidants that scavenge activated oxygen species include ascorbate acid and glutathione [11]. 

There is evidence that AM colonization could prevent or limit salt-induced oxidative stress in host plants [1,3,12,13]. However, reports on the response of antioxidant systems to stress in AM plants are inconsistent; increases, no change, or decreases in antioxidant enzyme activities have been reported in soybean [14,15], lettuce [16], and fenugreek [17] grown under stress conditions. The clarification of the possible role of antioxidants in the mycorrhizal effect may be helped by using genotypes differing in salt sensitivity.

Maize is a glycophyte and by definition, is salt-sensitive. To unravel the potential of AM fungi in alleviating salt toxicity in plants, a pot experiment using two maize genotypes with contrasting salt tolerance investigated the effect of AM fungus (*Funneliformis mosseae*) colonization on leaf water status, chlorophyll, gas exchange, chlorophyll fluorescence, oxidative damage to lipids, and antioxidant activity in maize under salt stress. Lower salt stress in AM maize plants was associated with improved plant growth and ion balance as already reported earlier [18]; this investigation would take it a step further to provide a full picture of AM-induced alleviation of salt stress in maize.

## 2. Materials and Methods

### 2.1. Growth Substrate

Loamy soil from a local maize/wheat rotation farm was sieved (<2.0 mm), mixed with fine sand (<2.0 mm) (1:1, *v/v*), and autoclaved at 0.11 MPa and 121 °C for 2 h. The mixed soil had 36.4 mg kg^−1^ N, 16.6 mg kg^−1^ available P, 171 mg kg^−1^ available K, 7.2 g kg^−1^ organic matter, and pH 6.9 (soil:water, 1:2.5, *w/v*). Plastic pots (200 mm in diameter, 160 mm height) were filled with 2.68 kg of the air-dried soil/sand mixture.

### 2.2. Biological Materials

Two maize (*Zea mays* L.) genotypes—salt-tolerant Jindan52 (JD52) and salt-sensitive Fushengyuan1 (FSY1)—were selected from preliminary studies that assessed root trait variability among 174 maize genotypes [19] and salinity tolerance in 20 selected genotypes [20] using a semi-hydroponic phenotyping system. selection of the two genotypes differing in salt tolerance were based on their ranking for salinity tolerance, in accordance with the reduction percent of shoot and root growth traits at 100 mM NaCl relative to the non-salt control, shoot Na^+^ and K^+^ contents, and shoot K^+^/Na^+^ ratios. Under salt stress, genotype JD52 had less reductions in growth, and lower shoot Na^+^ contents and higher shoot K^+^/Na^+^ ratios and was classified as salt-tolerant, while FSY1 was referred to as salt-sensitive [20].

The AM fungal species was *Funneliformis mosseae* (isolate BGC NM02A), propagated in pot cultures using white clover as a trap plant. A mixture of soil, mycelia, root fragments, and spores (14 spores per g soil) was used as AM inoculum.

### 2.3. Planting and Maintenance

Maize seeds were sterilized in a 10% H_2_O_2_ solution for 10 min, rinsed several times with sterile water, and then germinated in the dark on sterilized moist filter paper in Petri dishes at 28 °C. Five germinated seeds were transplanted in each pot on 5 September 2018. 

For each genotype, half the pots received 50 g of inoculum at sowing, just below the germinated maize seeds, as the mycorrhizal treatment (AM). The other half received the equivalent of autoclaved inoculum and its filtrate to reintroduce the same microflora without mycorrhizal fungi, as the non-mycorrhizal (NM) control. Fourteen days after sowing (DAS), the plants were thinned to two seedlings per pot.

Plants were established for 32 days before being treated with salt. For each genotype, half of the AM and NM plants were subjected to NaCl in gradual increments of 25 mM (i.e., 0.32g NaCl kg^–1^ soil) every 2nd day in a sufficient volume of de-ionized water to wet the soil to 80% field capacity (approximately 27.6% soil moisture content), until the required 100 mM concentration. The remaining AM and NM plants, as the non-salt controls, were watered to 80% field capacity with the same amount of water volume. The electrical conductivities were 0.2 and 8.2 dS m^-1^ for the growing substrate treated with 0 and 100 mM NaCl, respectively.

Plants were grown in greenhouse conditions with natural light, 50–75% relative humidity, and 24/15 °C day/night temperature. Pots were watered to weight daily, to maintain 80 ± 5% field capacity for the duration of the experiment. Each week, 50 mL of nutrient solution was supplied to each pot, containing (in µM): KNO_3_ (5000), Ca(NO_3_)_2_ (7200), KH_2_PO_4_ (1000), MgSO_4_ (4100), H_3_BO_3_ (46.3), MnCl_2_ (11.2), ZnSO_4_ (0.7), CuSO_4_ (0.32), H_2_MoO_4_ (0.1), FeEDTA (20). All of the pots were arranged in a completely randomized design with eight replicates, and randomly rearranged weekly during the experiment to reduce environmental effects. The experiment was assessed at 59 DAS.

### 2.4. Symbiotic Development

Small representative samples of fresh roots were taken instantly from each pot at harvest, gently washed, and fixed in 70% ethanol. Root samples were cut into 1-cm long pieces, followed by bleaching with 5% KOH (90 °C, 20 min), acidifying in 2% HCl (5 min, room temperature), and staining in 0.01% acid fuchsin (overnight, room temperature) [21]. The extent of root length colonization was estimated using the gridline intersect method.

### 2.5. Chlorophyll Content and Gas Exchange Parameters

Measurements were taken on the second youngest leaf of one plant from each pot (five plants per treatment) one day before harvest. Relative chlorophyll concentration was measured with a SPAD-502 plus chlorophyll meter (Spectrum, Aurora, IL, USA). Net photosynthetic rate, stomatal conductance, intercellular CO_2_ concentration, and transpiration rate were measured between 09:00 and 11:30 a.m. with a Li-6800 portable photosynthesis system (Li-COR, Lincoln, NE, USA). Water use efficiency was calculated as the ratio between the net photosynthetic rate and transpiration rate. Shoot water content was calculated according to our recent study [22].

### 2.6. Chlorophyll Fluorescence Parameters

Chlorophyll fluorescence was measured on the second youngest leaf of the same plant as above one day before harvest using a Li-6800 fluorometer (Li-COR, Lincoln, NE, USA). Plants were dark-adapted for 30 min, before measuring the dark-adapted minimal fluorescence (Fo), and maximal fluorescence (Fm) by applying a saturating actinic pulse of 8000 μmol m^−2^ s^−1^ for 1 s. The variable fluorescence (Fv = Fm − Fo) and maximum quantum yield of PSII photochemistry (Fv/Fm) were calculated from Fm and Fo.

The same leaf of each plant was used to determine light-adapted parameters. Steady-state fluorescence yield (Fs) was recorded in the light. A saturating actinic pulse of 8000 μmol m^−2^ s^−1^ for 1 s was applied to produce maximum fluorescence yield in the light-adapted state (Fm’). The actual quantum yield of PSII photochemistry (φPSII) and non-photochemical quenching (NPQ) were calculated as follows: φPSII = (Fm’ − Fs)/Fm’ and NPQ = Fm/Fm’ − 1.

### 2.7. Hydrogen Peroxide Content

The hydrogen peroxide content in leaves was measured following [23]. Fresh tissue (0.1 g) was homogenized in a cold mortar with 5 mL of 0.1% (*w/v*) trichloroacetic acid. The homogenate was centrifuged at 12,000× g for 20 min at 4 °C. The supernatant (0.5 mL) was mixed with 0.5 mL of 10 mM phosphate buffer (pH 7.0) and 1 mL of 1 M potassium iodide. The reaction mixture was incubated in the dark for 1 h, and absorbance at 390 nm was recorded. 

### 2.8. Membrane Lipid Peroxidation and Relative Electrolyte Leakage

Lipid peroxidation in leaves was estimated by measuring the concentration of malondialdehyde (MDA) [24]. Fresh leaves (0.1 g) were ground with 5 mL of 1% trichloroacetic acid (TCA) in a cold mortar, followed by centrifuging at 12,000× g for 10 min at 4 °C. The supernatant (0.5 mL) was mixed with 2 mL of 0.5% (*w/v*) thiobarbituric acid (TBA), and incubated at 100 °C for 30 min. After cooling, tubes were centrifuged at 5000 g for 10 min, and the supernatant used to measure absorbance at 532 nm (A_532_) and 600 nm (A_600_). The MDA concentration was calculated using an extinction coefficient of 155 mM^−1^ cm^−1^ by the following formula: MDA (μmol g^−1^ FW) = [(A_532_ − A_600_)/155] × 10^3^ × dilution factor.

For estimation of relative electrolyte leakage, fresh leaves (0.1 g) were placed in a test tube containing 10 mL of deionized water. The initial electrical conductivity (C1) was recorded after subjecting samples to incubation in a water bath at 25 °C for 30 min. The samples were then incubated at 100 °C for 30 min, cooled to room temperature, before recording the electrical conductivity (C2). The electrolyte leakage percentage was calculated as follows: [1 − C1/C2] × 100 [25].

### 2.9. Antioxidant Enzyme Activities

Enzymes were extracted using the method of [26] with slight modifications: fresh leaves (0.5 g) were homogenized in a cold mortar with 10 mL of 50 mM phosphate buffer (pH 7.8) containing 0.1 mM EDTA and 50 mg polyvinylpolypyrrolidone (PVPP). The homogenate was centrifuged in a refrigerated centrifuge at 12,000× g for 20 min at 4 °C. The supernatant was kept at −80 °C for subsequent assays. 

Total SOD activity was determined based on the ability of SOD to inhibit the photochemical reduction of nitroblue tetrazolium (NBT), as described by [27]. One unit of SOD was defined as the amount of enzyme causing a 50% inhibition of NBT reduction at 25 °C. The reaction mixture (3.3 mL) comprised 50 mM phosphate buffer (pH 7.8), 13 mM methionine, 75 μM NBT, 0.1 mM EDTA, 2 μM riboflavin, and 100 μL of enzyme extract. The riboflavin was added lastly. Tubes were shaken and then illuminated with fluorescent light. Controls and blanks were run in the same manner except enzyme and illumination, respectively. The reduction in NBT was measured at the absorbance of 560 nm.

CAT activity was assayed in a 2.5 mL reaction volume containing 50 mM phosphate buffer (pH 7.0), 10 mM H_2_O_2_, and 50 μL of enzyme extract. Adding the H_2_O_2_ initiated the reaction and the decrease in absorbance at 240 nm was recorded for 3 min to monitor the breakdown rate of H_2_O_2_ [28].

APX activity was determined according to the method described by [29]. The reaction mixture (2 mL) contained 50 mM phosphate buffer (pH 7.0), 0.1 mM EDTA, 0.5 mM ascorbate, 0.1 mM H_2_O_2_, and 100 μL of enzyme extract. The H_2_O_2_-dependent oxidation of ascorbate was monitored by a decrease in absorbance at 290 nm for 1 min.

The determination of GR activity followed the method of [30]. The reaction mixture (2 mL) contained 100 mM HEPES (pH 7.8), 1 mM EDTA, 0.5 mM oxidized glutathione, 0.2 mM NADPH, 3 mM MgCl_2_, and 200 μL of enzyme extract. The oxidation rate of NADPH was monitored by a decrease in the absorbance at 340 nm for 1 min. Two blanks, without enzyme and oxidized glutathione, respectively, were used as controls.

### 2.10. Antioxidant Molecules

The concentrations of reduced AsA and GSH were determined following the method of [31]. Fresh leaves (0.5 g) were powdered in liquid nitrogen and homogenized with 5 mL 5% (*w/v*) trichloroacetic acid. The homogenate was centrifuged at 12,000× g for 20 min at 4 °C, then the supernatant was used to determine AsA and GSH concentrations.

AsA was assayed in a reaction mixture (3 mL) containing 100 mM phosphate buffer (pH 7.0), 10% (*w/v*) trichloroacetic acid, 44% (*w/v*) H_3_PO_4_, 4% (*w/v*) 2,2′-bipyridyl, 3% (*w/v*) FeCl_3_, and 0.5 mL of supernatant. The mixture was incubated at 37 °C for 1 h. After cooling to room temperature, the absorbance was read at 525 nm. The AsA concentration was calculated according to a standard curve prepared with ascorbic acid. GSH was determined in a reaction mixture (2.5 mL) containing 100 mM phosphate buffer (pH 7.0), 0.6 mM 5,5′-dithiobis (2-nitrobenzoic acid), and 1 mL of supernatant. The absorbance of the mixture was recorded at 412 nm. The GSH concentration was calculated from a standard curve of glutathione.

### 2.11. Statistical Analysis

Experimental data were processed with the SPSS 17.0 statistical program (IBM, USA). The analysis of variance (ANOVA) of General Linear Model multivariate analysis was performed considering maize genotypes, NaCl, and AM fungi as independent factors. Tukey’s multiple comparison test was used to estimate differences between individual means at *p* ≤ 0.05.

## 3. Results

### 3.1. Symbiotic Development

Both maize genotypes had around 90% of mycorrhizal root length without NaCl, and the NaCl treatment did not significantly change the extent of mycorrhizal colonization. No mycorrhizal colonization was observed in uninoculated plants.

### 3.2. Leaf Water Status

**Figure 1:**
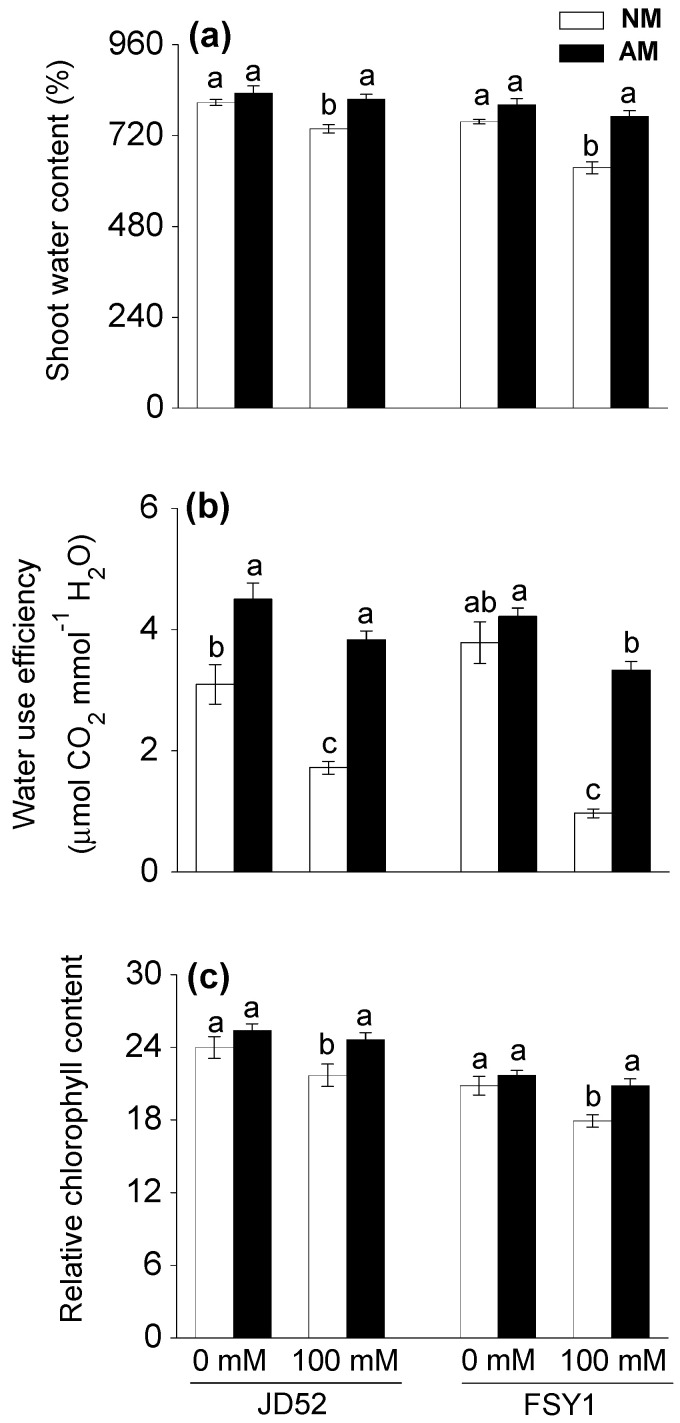
Relative water content (**a**), water use efficiency (**b**), and relative chlorophyll content (**c**) in two maize genotypes (JD52 and FSY1) inoculated with *Funneliformis mosseae* (AM) or without inoculation (NM) in 0 and 100 mM NaCl treatments assessed at 59 DAS. For each trait, bars with different letters indicate significant differences (*p* ≤ 0.05).

Relative to the non-salt control, the 100 mM NaCl treatment significantly decreased shoot water content in NM plants by 9% and 16% in JD52 and FSY1, respectively (Table 1, Figure 1a). The corresponding decreases in the AM plants were not significant. Shoot water content did not significantly differ between NM and AM plants in both genotypes at 0 mM NaCl; however, the 100 mM NaCl treatment increased shoot water content in AM plants, by 11% (JD52) and 21% (FSY1) relative to the respective NM plants.

The 100 mM NaCl treatment significantly decreased water use efficiency of NM plants by 44% and 74% in JD52 and FSY1, respectively, relative to the non-salt control (Table 1, Figure 1b). The corresponding decreases in AM plants were only 15% and 21%. At 0 mM NaCl, the water use efficiency of AM plants was 45% higher than NM plants for JD52, but not significantly different for FSY1. At 100 mM NaCl, AM plants had 123% (JD52) and 244% (FSY1) higher water use efficiency than their respective NM plants.

### 3.3. Chlorophyll

For NM plants, the 100 mM NaCl treatment significantly decreased relative chlorophyll content by 10% and 14% in JD52 and FSY1, respectively, relative to the non-salt control (Table 1, Figure 1c). For AM plants, the corresponding decreases were only 3% and 4%. At 0 mM NaCl, AM plants and NM plants had similar relative chlorophyll contents in both genotypes. At 100 mM NaCl, AM plants had 14% (JD52) and 16% (FSY1) higher relative chlorophyll contents than the respective NM plants.

### 3.4. Gas Exchange

**Figure 2:**
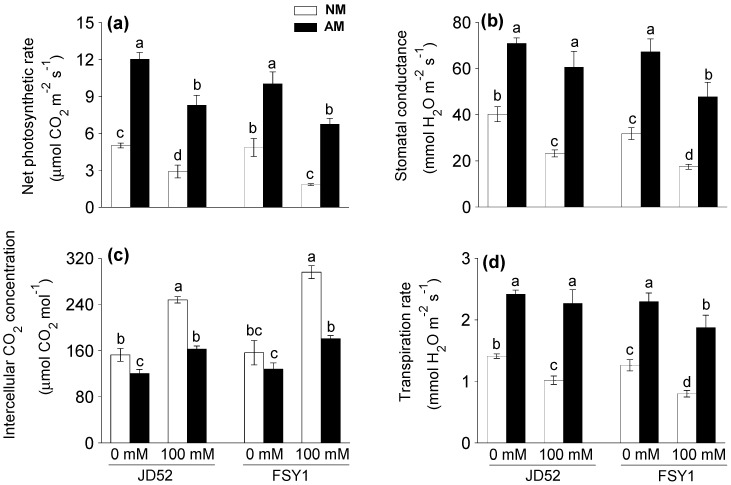
Net photosynthetic rate (**a**), stomatal conductance (**b**), intercellular CO_2_ concentration (**c**), transpiration rate (**d**) in two maize genotypes (JD52 and FSY1) inoculated with *Funneliformis mosseae* (AM) or without inoculation (NM) in 0 and 100 mM NaCl treatments assessed at 59 DAS. For each trait, bars with different letters indicate significant differences (*p* ≤ 0.05).

Salinity significantly decreased net photosynthetic rate with or without AM inoculation (Table 1, Figure 2a). Relative to the non-salt control, the 100 mM NaCl treatment reduced the net photosynthetic rate in NM plants by 42% (JD52) and 62% (FSY1). The corresponding reductions in AM plants were 31% and 33%. AM inoculation significantly increased net photosynthetic rate by 140% (JD52) and 107% (FSY1) at 0 mM NaCl, and 186% (JD52) and 267% (FSY1) at 100 mM NaCl.

For NM plants, the 100 mM NaCl treatment reduced stomatal conductance by 42% (JD52) and 45% (FSY1), relative to the non-salt control (Table 1, Figure 2b). For AM plants, the corresponding reductions were 14% and 29%. AM inoculation significantly increased stomatal conductance by 76% (JD52) and 111% (FSY1) at 0 mM NaCl, and 161% (JD52) and 175% (FSY1) at 100 mM NaCl.

The 100 mM NaCl treatment reduced the transpiration rate in NM plants by 28% (JD52) and 37% (FSY1), relative to the non-salt control (Table 1, Figure 2d). The corresponding reductions in AM plants were 6% and 19%. AM inoculation significantly increased transpiration rate by 72% (JD52) and 82% (FSY1) at 0 mM NaCl, and 122% (JD52) and 134% (FSY1) at 100 mM NaCl.

Intercellular CO_2_ concentration increased significantly after salinity application (Table 1, Figure 2c). Relative to the non-salt control, the 100 mM NaCl treatment increased the intercellular CO_2_ concentration in NM plants by 63% (JD52) and 89% (FSY1). The corresponding increases in AM plants were 35% and 41%. Intercellular CO_2_ concentration decreased with AM inoculation, by 21% (JD52) and 18% (FSY1) at 0 mM NaCl, and 34% (JD52) and 39% (FSY1) at 100 mM NaCl.

### 3.5. Chlorophyll Fluorescence

**Figure 3:**
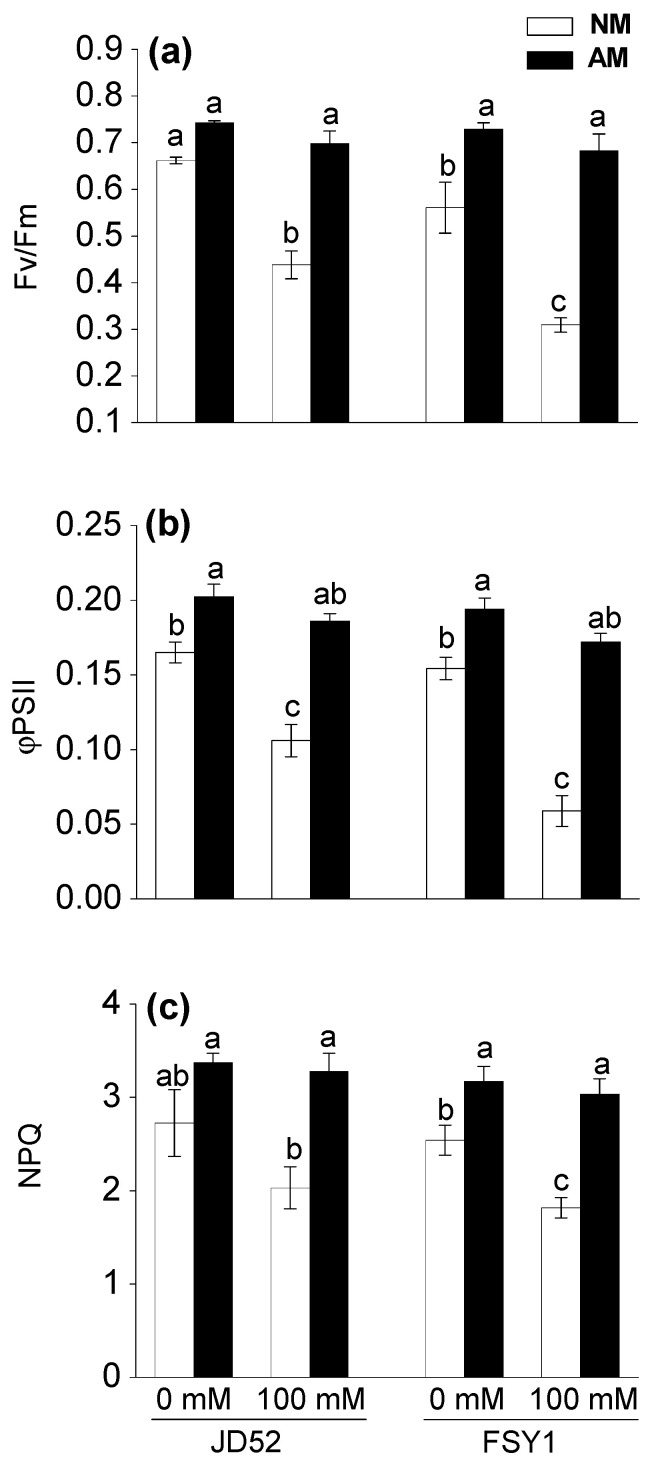
Maximum quantum yield of PSII photochemistry (Fv/Fm) (**a**), actual quantum yield of PSII photochemistry (φPSII) (**b**), and non-photochemical quenching (NPQ) (**c**) in two maize genotypes (JD52 and FSY1) inoculated with *Funneliformis mosseae* (AM) or without inoculation (NM) in 0 and 100 mM NaCl treatments assessed at 59 DAS. For each trait, bars with different letters indicate significant differences (*p* ≤ 0.05).

For NM plants, the 100 mM NaCl treatment significantly reduced Fv/Fm by 34% and 45% in JD52 and FSY1, respectively, relative to the non-salt control (Table 1, Figure 3a). For AM plants, the corresponding reductions were not significant and similar for both genotypes (6%). Fv/Fm increased with AM inoculation, by 12% (JD52) and 30% (FSY1) at 0 mM NaCl, and 59% (JD52) and 121% (FSY1) at 100 mM NaCl.

For NM plants, the 100 mM NaCl treatment significantly reduced φPSII by 36% (JD52) and 62% (FSY1), relative to the non-salt control (Table 1, Figure 3b). For AM plants, the corresponding reductions were only 8% and 11%. AM inoculation significantly increased φPSII by 23% (JD52) and 26% (FSY1) at 0 mM NaCl, and 75% (JD52) and 193% (FSY1) at 100 mM NaCl. 

Relative to the non-salt control, the 100 mM NaCl treatment reduced the NPQ in NM plants by 26% (JD52) and 29% (FSY1), respectively (Table 1, Figure 3c). For AM plants, the 100 mM NaCl treatment reduced NPQ slightly to a similar level in both genotypes—3% (JD52) and 4% (FSY1), relative to the non-salt control. NPQ increased with AM inoculation, by 24% (JD52) and 25% (FSY1) at 0 mM NaCl, and 62% (JD52) and 67% (FSY1) at 100 mM NaCl.

### 3.6. Hydrogen Peroxide Accumulation

**Figure 4:**
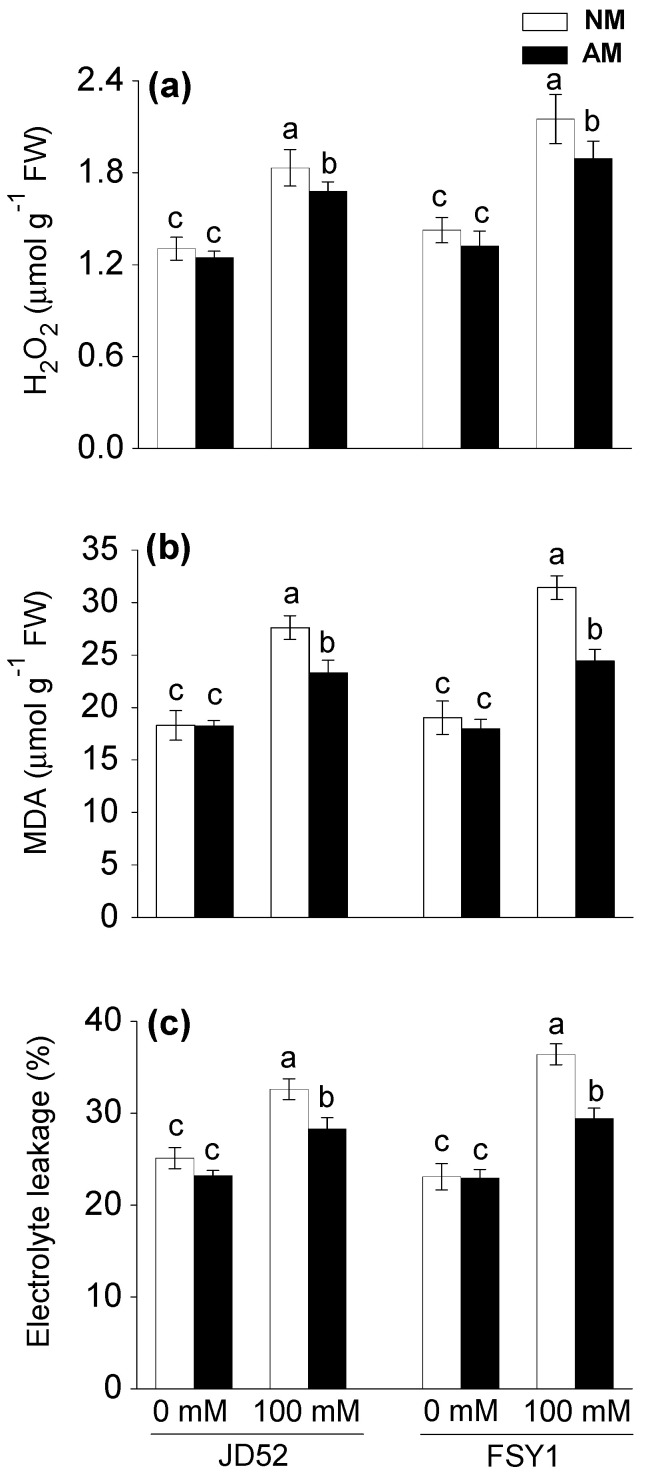
Hydrogen peroxide content (**a**), lipid peroxidation (**b**), and electrolyte leakage (**c**) in two maize genotypes (JD52 and FSY1) inoculated with *Funneliformis mosseae* (AM) or without inoculation (NM) in 0 and 100 mM NaCl treatments assessed at 59 DAS. For each trait, bars with different letters indicate significant differences (*p* ≤ 0.05).

Salinity significantly increased H_2_O_2_ production in maize plants with or without AM inoculation (Table 1, Figure 4a). For NM plants, the 100 mM NaCl treatment increased H_2_O_2_ concentration by 40% (JD52) and 51% (FSY1), relative to the non-salt control. For AM plants, the corresponding increments were 35% and 43%. At 100 mM NaCl, AM inoculation significantly decreased H_2_O_2_ concentration by 8% and 12% in JD52 and FSY1, respectively.

### 3.7. Oxidative Damage to Lipids and Relative Electrolyte Leakage

The oxidative damage to lipids and electrolytic leakage significantly increased in maize plants as a consequence of salinity (Table 1, Figure 4b,c). For NM plants, the 100 mM NaCl treatment increased lipid peroxidation by 51% and 65% in JD52 and FSY1, respectively, relative to the non-salt control. The corresponding increments in AM plants were 28% and 36%. The AM and NM plants of both genotypes had similar levels of lipid peroxidation at 0 mM NaCl. At 100 mM NaCl, AM plants had 16% (JD52) and 22% (FSY1) less lipid peroxidation than the respective NM plants.

The 100 mM NaCl treatment increased electrolytic leakage in NM plants by 30% and 58% in JD52 and FSY1, relative to the non-salt control. The corresponding increases in AM plants were 18% and 28%. At 0 mM NaCl, AM and NM plants had similar electrolytic leakage in both genotypes. At 100 mM NaCl, AM plants had 13% (JD52) and 19% (FSY1) lower electrolytic leakage than the respective NM plants.

### 3.8. Antioxidant Enzyme Activities

**Figure 5:**
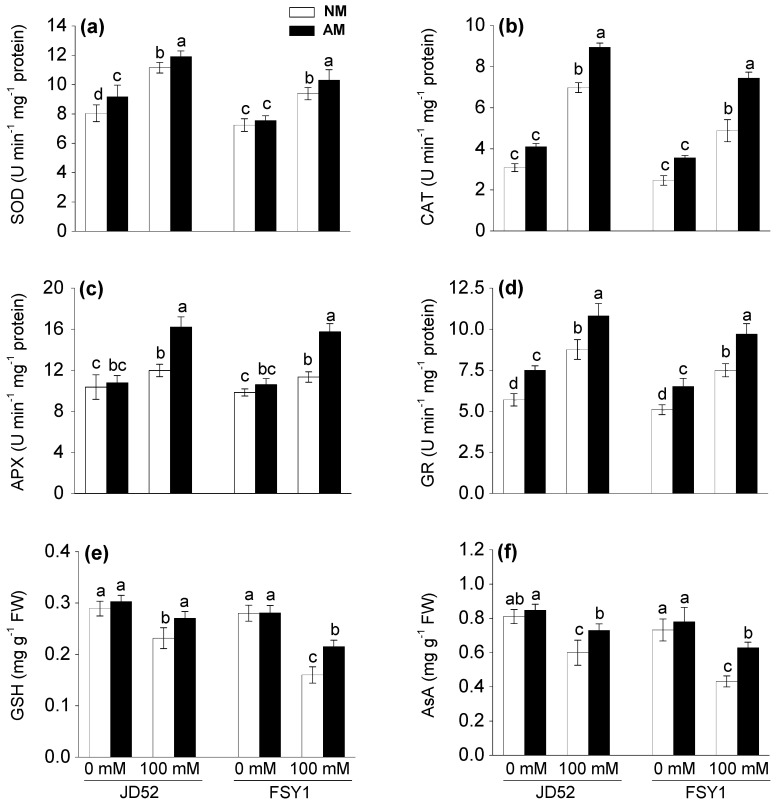
Superoxide dismutase (SOD) (**a**), catalase (CAT) (**b**), ascorbate peroxidase (APX) (**c**), and glutathione reductase (GR) (**d**) activities, and reduced glutathione (GSH) (**e**) and ascorbate (AsA) (**f**) contents in two maize genotypes (JD52 and FSY1) inoculated with *Funneliformis mosseae* (AM) or without inoculation (NM) in 0 and 100 mM NaCl treatments assessed at 59 DAS. For each trait, bars with different letters indicate significant differences (*p* ≤ 0.05).

SOD, CAT, APX, and GR activities in both genotypes significantly increased when cultivated under salinity, with and without AM inoculation (Table 1, Figure 5a–d). For JD52 at 100 mM NaCl, the SOD, CAT, APX, and GR activities of NM plants increased by 39, 126, 16, and 54%, respectively, relative to the non-salt control. The corresponding increases in AM plants were 30, 118, 50, and 44%. For FSY1 at 100 mM NaCl, relative to the non-salt control, the increases in antioxidant activities in NM plants were less than those in JD52, i.e., 30, 98, 15, and 47%, respectively. The corresponding increases in AM plants of FSY1 were 36%, 109%, 48%, and 49%. No significant differences in SOD, CAT, APX, and GR activities occurred between AM and NM plants in either genotype at 0 mM NaCl, except for SOD activity in JD52 and GR activity in both genotypes, where AM plants had higher activity than NM plants. In contrast, at 100 mM NaCl, AM plants had significantly higher SOD, CAT, APX, and GR activities than NM plants, being 7, 28, 35, and 23% higher in JD52, and 10, 52, 39, and 29% higher in FSY1, respectively.

### 3.9. Antioxidant Molecules

The GSH and AsA contents decreased in both genotypes by NaCl application, regardless of AM inoculation, expect for GSH content in AM plants of JD52, which remained similar to the control conditions (Table 1, Figure 5e,f). For JD52 at 100 mM NaCl, the GSH and AsA contents decreased in NM plants by 20 and 26%, respectively, relative to the non-salt control. The corresponding decreases in AM plants were 11 and 14%. For FSY1 at 100 mM NaCl, the GSH and AsA contents decreased in NM plants by 43 and 41%, respectively, relative to the non-salt control. The corresponding decreases in AM plants were 23 and 19%. At 0 mM NaCl, the GSH and AsA contents did not significantly differ between AM and NM plants in either genotype. However, at 100 mM NaCl, AM plants had significantly higher GSH and AsA contents than NM plants, being 17 and 22% higher in JD52, and 35 and 46% higher in FSY1.

## 4. Discussion

Salinity deters photosynthetic CO_2_ assimilation due to the reduced stomatal conductance induced by the osmotic component of salt stress. In the second phase, salinity might affect photosynthesis through biochemical and photochemical disruption [32]. The lack of CO_2_ assimilation causes buildup of excess energy and, if not quenched, may lead to an excess of electrons accumulated from the photochemical phase in thylakoid membranes. This could trigger over-reduction of the reaction center of PSII and thus damage to the components of photosynthetic apparatus [33], such as membrane integrity, which further attenuates photosynthetic ability. In this study, salt stress decreased the gas exchange parameters (except intercellular CO_2_ concentration), and disruption of these processes was correlated with genotypic differences in the response to salinity, FSY1 being more affected than JD52 (Table 1, Figure 2). The reduced shoot water content in leaves (Figure 1a) under saline conditions induced stomatal closure, causing a decline in CO_2_ availability and photosynthesis rate. However, AM maize plants, especially JD52, maintained a higher net photosynthetic rate, stomatal conductance and transpiration rate, and lower intercellular CO_2_ concentration than NM plants under both non-saline and saline conditions (Figure 2). The increased gas exchange in AM plants has been associated with improved water uptake and translocation [6,34], and also plant hormonal levels as observed in other studies [35,36]. Our results showed that salt-stressed AM plants, especially JD52, had higher shoot water content and water use efficiency than the salt-stressed NM plants (Figure 1a,b). Indeed, the improvement of water status due to AM symbiosis plays an indirect role in increasing the photosynthetic capacity [6].

An increase in intercellular CO_2_ concentration under stress conditions indirectly indicates the disruption of photosynthetic apparatus, since salt stress induces a decrease in stomatal conductance and inactivation of enzymes that can facilitate CO_2_ to accumulate in intercellular areas [37,38]. In contrast, the higher photosynthesis under decreased intercellular CO_2_ concentration in AM plants subjected to salinity (Figure 2c) implies that CO_2_ is used more efficiently with AM symbiosis [6,39]. Thus, these results illustrate protection of photosynthetic machinery and enhanced CO_2_ fixation in maize leaves by AM colonization, and the effects were more pronounced in FSY1 than JD52.

Chlorophyll is a crucial component for plant photosynthesis and closely reflects the photosynthetic capacity of a plant [40]. The results of our study showed that AM inoculation increased the chlorophyll concentration in maize leaves (Figure 1c). Enhanced chlorophyll content has been reported for other AM-inoculated plants including wheat, rice, and sesbania, subjected to salinity [2,39,41]. 

The processes of photosystem II have widely been used to study plant responses and adaption to a variety of stresses [42]. Chlorophyll fluorescence parameters accurately reflect the photosynthetic processes [43]. The ratio of Fv/Fm reflects the potential quantum efficiency of PSII photochemistry and is served as an indicator of plant photosynthetic performance [44]. This study showed that Fv/Fm, along with φPSII, was significantly higher in the leaves of AM plants than that in NM plants under saline conditions, especially JD52 (Figure 3a,b). In the meantime, NPQ in AM plants, especially JD52, was significantly higher than NM plants (Figure 3c). Normally, NPQ quantifies the quantum yield of non-photochemical quenching, and is a measure of the efficiency of heat dissipation [45,46]. An increase in NPQ acts as a mechanism to protect the leaf from light-induced damage [45,46]. Thus, our data imply that AM plants, especially JD52, had higher photochemical efficiency and a better ability to protect leaves from light injury; thereby, in addition to the increase in stomatal conductance and water status, AM plants might improve net photosynthetic rate by protecting the photochemical processes of photosystem II (Figure 2 and Figure 3) when subjected to salinity. Although under salt stress the leaf chlorophyll content and associated fluorescence properties decreased more in FSY1 than JD52 (Figure 1c, Figure 3), the positive effects of *F. mosseae* inoculation were more expressed in FSY1 than JD52.

The strengthened photosynthetic processes in AM plants could inhibit photorespiration and result in a decline in ROS production. Indeed, we observed that salt-stressed AM plants, especially JD52, had consistently lower accumulation of H_2_O_2_ than NM plants (Figure 4a). While salinity increased production of H_2_O_2_ and led to membrane lipid peroxidation (indicated by MDA content) and ion leakage, especially in salt sensitive genotype FSY1, the AM plants, particularly tolerant genotype JD52, exhibited less oxidative damage to the lipids and better membrane integrity than NM plants (Figure 4). Amelioration of oxidative damage to membrane by AM symbiosis was also reported in soybean [15] and wheat [47] under salinity stress.

The oxidation of membrane lipids is an indication of excess free radical production related to oxidative stress [48], and crop species or genotypes with lower leaf lipid peroxidation and ion leakage are considered more tolerant to salt stress [49,50,51]. In our study, the increase or degree of NaCl-induced oxidative damage in leaves was less in JD52 than FSY1 (Figure 4b,c); this observation demonstrated genotypic variation in tolerance to salinity between the two tested maize genotypes and the higher salt tolerance in JD52 compared to FSY1.

Plant salt tolerance is associated with induction of the antioxidant defense system and prevention of oxidative damage [52]. In this study, antioxidant enzyme activities increased on exposure to salinity in both genotypes regardless of AM inoculation (Figure 5a–d). However, the magnitude of increase was greater in NM plants of JD52 than FSY1, but varied between AM plants of both genotypes. Genotype JD52 had higher antioxidant enzyme activities than FSY1 regardless of AM inoculation, which confirms an ability for antioxidant defenses in genotype differences to cope with oxidative stress [49,50] and again supporting the relative tolerance of JD52. 

The better performance of photochemical processes in AM plants may have contributed to a lower production of ROS and lipid peroxidation rate, as discussed formerly. However, the activation of antioxidant systems could have accounted likewise. Our observations showed that AM inoculation further increased antioxidant enzyme activities in maize plants under salt stress (Figure 5a–d), which enabled quicker scavenging of ROS and prevented oxidative stress. SOD plays a key role in maintaining cell membrane stability by catalyzing the conversion of superoxide radicals. Several studies have demonstrated that AM colonization enhances SOD activity in plants grown under salinity stress [14]. The enhanced SOD activity observed in AM plants, relative to NM plants (Figure 5a), indicates that these fungi increase the capacity of maize plants to scavenge superoxide radicals and hence protecting membrane integrity and electron transport. The resultant generation of H_2_O_2_ is eliminated by CAT or APX in the ascorbate-glutathione cycle, where GR, GSH, and AsA are necessary components for ROS detoxification [8]. Increased CAT and APX activities that lead to the acceleration of H_2_O_2_ breakdown prevent cellular oxidative damage. GR is a key component of the ascorbate-glutathione cycle, where the net electron flow is from NADPH to H_2_O_2_ resulting in conversion of H_2_O_2_ into H_2_O [11]. Elevated GR activity leads to increased production of reduced glutathione, which acts as electro donor during the conversion of dehydroascorbate into AsA, and AsA acts as an electron donor in conversion of H_2_O_2_ to H_2_O and O_2_ [11]. Upregulation of GR keeps higher NADP^+^/NADPH and GSH/GSSH ratios to reduce the formation of ROS and hence maintain photosynthetic electron transport [11]. In the present study, AM plants, especially JD52, had higher activities of antioxidant enzymes (SOD, CAT, APX, GR) than NM plants, implying a better ROS scavenging system in them. These results agree with previous observations in mycorrhizal plants under salinity, such as pigeonpea [53], *Pennisetum glaucum* [54], and wheat [47]. However, the extent of effects of *F. mosseae* inoculation on these parameters varied between the tested genotypes grown under salt stress, being greater in FSY1 than in JD52.

Salt stress reduced GSH and AsA contents in both genotypes regardless of AM inoculation, especially in FSY1 (Figure 5e,f), as reported by Umar et al. [55] in *Brassica campestris*. However, AM inoculation increased GSH and AsA contents in both genotypes under salt stress. The increase in GR activity and GSH and AsA contents in AM maize plants could strengthen the antioxidant system for ROS scavenging, similarly observed in fenugreek [17]. Analogous to the antioxidant enzymes, AM plants, especially JD52, had higher contents of GSH and AsA than NM plants, and the inoculation effects of *F. mosseae* were more evident in FSY1 than JD52 under salt stress. Overall, the ameliorative effects of AM colonization on the studied physiological parameters were greater in salt-sensitive genotype FSY1 than salt-tolerant genotype JD52, indicating genotypic differences in the amelioration of salt stress by AM fungi.

## 5. Conclusions

Salinity negatively affected photosynthetic processes by inducing the inhibition of photosynthesis and associated disintegration of photosynthetic membrane caused by oxidative stress, especially in salt-sensitive genotype FSY1. AM plants, especially salt-tolerant genotype JD52, exhibited lower oxidative damage in photosynthetic machinery, and higher capacity and efficiency for CO_2_ assimilation, however, AM inoculation ameliorated salinity-induced toxic effects more in FSY1, by upregulating the antioxidant defense systems and simultaneously preventing oxidative injury of membrane and enhancing activity and efficiency of photosynthesis. Data reported in this study were acquired from a controlled experiment with thoughtful designs and careful implementation, and further study will validate these findings in different environments involving more genotypes and/or isolates.

## Figures and Tables

**Table 1:** Significance of three-way ANOVA for 19 physiological and biochemical traits in two maize genotypes (JD52 and FSY1) inoculated with *Funneliformis mosseae* (arbuscular mycorrhizal) (AM) or without inoculation (non-mycorrhizal) (NM) in 0 and 100 mM NaCl treatments assessed at 59 days after sowing (DAS).

Trait	G	S	AM	G × S	G × AM	S × AM	G × S × AM
Shoot water content	***	***	***	n.s.	*	***	n.s.
Water use efficiency	n.s.	***	***	**	n.s.	***	n.s.
Relative chlorophyll content	***	***	***	n.s.	n.s.	n.s.	n.s.
Net photosynthetic rate	**	***	***	n.s.	n.s.	n.s.	n.s.
Stomatal conductance	*	***	***	n.s.	n.s.	n.s.	n.s.
Intercellular CO_2_ concentration	*	***	***	n.s.	n.s.	***	n.s
Transpiration rate	*	***	***	n.s.	n.s.	n.s.	n.s.
Fv/Fm	*	***	***	n.s.	*	***	n.s.
φPSII	***	***	***	n.s.	n.s.	***	n.s.
NPQ	n.s.	*	***	n.s.	n.s.	n.s.	n.s.
H_2_O_2_	***	***	***	*	n.s.	n.s.	n.s.
MDA	***	***	***	**	*	***	n.s.
Electrolyte leakage	n.s.	***	***	***	n.s.	***	**
SOD	***	***	***	n.s.	n.s.	n.s.	n.s.
CAT	***	***	***	***	n.s.	***	n.s.
APX	n.s.	***	***	n.s.	n.s.	***	n.s.
GR	***	***	***	n.s.	n.s.	n.s.	n.s.
GSH	***	***	***	***	n.s.	**	n.s.
AsA	***	***	***	n.s.	n.s.	***	n.s.

The sources of variation were genotype (G), salt treatment (S), AM inoculation (AM), and their interactions (G × S, G × AM, S × AM, G × S × AM). * *p* ≤ 0.05; ** *p* ≤ 0.01; *** *p* ≤ 0.001; n.s., not significant. Abbreviations: Fv/Fm, maximum quantum yield of PSII photochemistry; φPSII, actual quantum yield of PSII photochemistry; NPQ, non-photochemical quenching; H_2_O_2_, hydrogen peroxide; MDA, malondialdehyde; SOD, superoxide dismutase; CAT, catalase; APX, ascorbate peroxidase; GR, glutathione reductase; GSH, glutathione; AsA, ascorbate.
